# Store owners as potential agents of change: energy drinks in the interior of Alaska

**DOI:** 10.1080/22423982.2017.1400362

**Published:** 2017-11-20

**Authors:** Janet M. Wojcicki, Peter de Schweinitz

**Affiliations:** ^a^ Department of Pediatrics, University of California San Francisco, San Francisco, CA, USA; ^b^ Chief Andrew Isaac Health Center, Medical Services, Tanana Chiefs Conference, Fairbanks, AK, USA

**Keywords:** Public health interventions, energy drinks, obesity, Indigenous Health, Alaska Native Health, rural Alaska

## Abstract

Childhood obesity disproportionately impacts disadvantaged communities, including Alaska Native children. In part, lack of access to fresh fruits and vegetables and over consumption of sugar sweetened beverages including energy drinks contribute to excessive weight gain in Alaska Native youth. This commentary reports the possibility of storeowners and workers partnering with community members to limit sales of nutrient-poor energy drinks through point-of-sale counselling in rural communities in the interior of Alaska. This model of intervention may be useful to implement in areas where there are limited health workers or others that can serve as health educators. This study reports preliminary evidence from rural Alaska and from other Arctic communities that store workers may effectively improve community health status by limiting or promoting specific products. Storeowners or workers may be helpful partners in the fight against childhood obesity as they are present at the point of sale of high-risk beverages to Alaska Native youth.

## Energy drinks in rural Alaska

American Indian and Alaska Native children have the highest prevalence of obesity among all US racial and ethnic groups. A national study of pre-school-age children in 2005 found that 31.2% of American Indian and Alaska Native children were obese at 4 years of age [1]. Alaska Native children have an even higher prevalence than what is reported nationally for American Indians and Alaska Natives as a group. We reported that 42.4% of 3-year-old Alaska Native children were obese in a statewide survey in 2008–2009 and an additional 24.9% were overweight [2]. Alaska Native adolescents are also significantly more likely than white adolescents to be obese, with 16% of Alaska Native Alaskan high school students considered obese, compared to 10% of white Alaskan high school students [3]. Energy drinks such as Monster Energy, Red Bull and Rockstar may be one significant cause of this epidemic, particularly in adolescents.

Energy drinks, which typically contain high concentrations of both caffeine and sugar, contribute not only to obesity, but also to diabetes mellitus type 2, cardiovascular disease and neurologic events. Concluding that caffeine and other (addictive) stimulant substances have no place in the healthy diet of children or adolescents, the American Academy of Pediatrics recommends that children not drink any energy drinks [4]. Previous work with Alaska Native and non-Native college students has also found that use of energy drinks in adolescence is associated with hazardous alcohol consumption, in addition to obesity [5].

Our work in rural Alaska suggests that adult residents of Alaskan communities want to decrease the energy drink consumption in youth. In our work in a rural, interior Alaskan village during summer 2014, we found that 29% of children and 52% of adolescents reported drinking energy drinks or sports drinks at a minimum of once per week, although we did not differentiate between energy and sports drinks in our reporting [6]. In focus groups, residents lamented that many of the young people wanted to buy energy drinks, which were seen as flooding the local stores. What may be surprising is that many of the storeowners and managers themselves spoke out against energy drink consumption in youth, and some already limit the sales of such products to youth in their villages. We believe that storeowners and managers can and should be engaged as health advocates to help lower energy drink consumption in rural areas.

## Restriction of energy drinks

The desire to limit energy drink consumption in youth is already present in many Native American communities. In the lower 48 states, large American Indian populations have policies in place to ban energy drinks. As part of its Wellness Policy, the Chemawa Indian School [7] prohibits energy drinks because of their high caffeine and sugar content. The T’iis Ts’ozi B’Olta Crownpoint Community School Wellness Policy specifically advises that anyone who brings energy drinks to school will be suspended [8]. Likewise, some school districts in the interior of Alaska explicitly ban energy drinks as part of their school Wellness Policy [9], while others do not have explicit bans in their student handbooks (e.g. Tanana, Galena) [10,11]. We presume that school-based policies dramatically reduce energy drink consumption during school hours; however, the hours per year a child is out of school vastly outnumber those in school. Alaska Native communities have a history of regulating access to alcohol due to the local option law passed in 1980 [12]. Although not legally mandated or enforced, storeowners could chose to not stock or limitedly stock energy drinks in small towns.

## Store-based interventions

Store-based nutrition interventions have been shown to improve dietary intake and eating behaviours in hard to reach populations and are a promising approach to address obesity [13,14]. There is recent evidence that, by changing the physical structure, product display and product selection of the store (e.g. in this case, reduced volume of energy drinks for sale), store-based interventions can influence the consumption patterns of clients and that these changes can be met with a positive reception from store owners, due to increased sales [15–17].

A variety of approaches to in-store obesity reduction and healthy diet promotion have been successful. These interventions have focused on healthy cooking and providing recipes and promotional point of purchase items such as posters for healthy nutrition. A recent study found that social messages on grocery cart placards increase produce spending [18]. Point of purchase nutrition information on vegetables can result in increases in vegetables sales [19,20]. Critical to these efforts has been the support of storeowners and managers, who have proven themselves to be valuable partners in improving visibility of healthier food options and making recipes and nutrition-related information available [21].

Previous store-based interventions in rural communities and with youth in American Indian communities have also demonstrated improvements in eating habits and obesity [13,22,23]. A store-based intervention in the Navajo Nation of American Indians found that greater exposure to the intervention was associated with significantly reduced body mass index (BMI) [22]. Other store-based interventions in isolated circumpolar, indigenous communities including Inuit and Inuvialuit [24] have been successful in partnering with local stores and promoting sugar-free and low fat food alternatives in the local grocery store [25]. While interventions in Arctic communities face issues of remoteness and climate that may impact the increased provision of fresh produce and other goods [24], specific interventions such as targeting energy drink or soda sales should not be impacted by the location or geography of Arctic communities.

## Curbing youth energy drink consumption in rural Alaska

While there is a long history of store-based interventions in American Indian and First Nations communities [23,25–27], these types of intervention are yet to be developed with Alaska Native populations. In some areas, working with store owners is still considered challenging because of the inherent conflict of interest for the store owner, who needs to run a business, and because of the common perception that customers may not want to purchase healthier products [28]. As the village store can play a central role in the community in rural Alaska, however, and because storeowners and managers are commonly well-known, long-term members of the community with vested interests in the health of their customers (who are often close and extended relatives), we believe that store owners are critical to successful reduction of energy drink consumption in youth in interior Alaska. In rural Alaska, stores are often run and owned by village Native corporations, numbering over 200 [29].

Although we acknowledge the existence of concerns about the potential of decreasing store profits made from the sale of energy drinks to children, our experience working in rural Alaska and discussing potential interventions with store owners suggests that many are willing to take measures to promote healthy beverage consumption, especially in the interests of children. Indeed, in 2016, we found that store owners and managers in rural areas of interior Alaska that had boarding schools such as Nenana were *already* partnering with the local stores to limit the sale of energy drinks to children and adolescents. Specifically, although energy drinks were not banned from local stores, store owners and workers attempted to dissuade adolescents from purchasing these drinks and encourage them to choose healthier options. With regard to children under 10, we have been told that, in some villages, store owners and workers refuse to sell energy drinks to children and/or counsel them that their parents will be told.

As our group has been researching the feasibility of conducting interventions in neighbourhood stores in rural Alaska, we have been advised in a few of the communities such as Huslia and Fort Yukon that store owners have already, independently partnered with school officials to limit the consumption of energy drinks for adolescents through point of sale counselling. While these type of interventions may not prove successful in larger urban or suburban areas, in small rural Alaskan towns, there may be easier partnerships between non-traditional partners—storeowners/workers, teachers and parents.

It appears that interior Alaskan store owners and managers, who themselves are typically members of these tight-knit communities, are invested in the health of the next generation. Although barriers exist, our experiences in interior Alaska and the effectiveness of programmes in the lower 48 states suggest that storeowners and managers can be brought in as partners to redress the obesity and metabolic disease epidemic. Community-based research projects that enlist store owners and store managers to develop effective programmes of store-based intervention, including energy drink sales restriction, should be developed to prevent more children from developing unhealthy patterns of consumption.
